# Patient perspectives on the management of COPD and Type 2 Diabetes in general practice: an interview study

**DOI:** 10.1186/s12875-022-01787-8

**Published:** 2022-07-14

**Authors:** Kim Lee, Signe Beck Titlestad, Birgitte Nørgaard, Niels Bentzen, Jens Søndergaard, Michael Marcussen

**Affiliations:** 1 Department of Public Health, Research Unit of General Practice, University of Southern Denmark, Odense, Denmark; 2 Department of Research, University College South Denmark, Esbjerg, Denmark; 3 Department of Public Health, Research Unit of User Perspective, University of Southern Denmark, Odense, Denmark

**Keywords:** General practice, Qualitative research, Organisational Changes, Primary Care

## Abstract

**Background:**

The Danish healthcare system has undergone fundamental organisational changes. In recent years, treatment of most patients with chronic obstructive pulmonary disease (COPD) and type 2 diabetes (T2D) in Denmark has been transferred from specialised hospitals to general practices, and only the most complicated cases are treated at hospital outpatients clinics or are admitted. This transfer aimed to reduce costs without compromising quality of care and ensure that the treatment was managed by general practitioners (GPs) who had personal knowledge of the patient. In this paper, we explore patients’ perceptions of the quality of care provided by their GPs.

**Methods:**

A qualitative research study was conducted with semi-structured interviews of 24 informants; nine were diagnosed with COPD and 15 were diagnosed with T2D. Snowball sampling was used for recruitment. Data were analysed using systematic text condensation.

**Results:**

The interviews revealed four main themes:

1) The informants perceived the quality of their treatment in general practice to be high due to their personal relationship with their GPs.

2) The informants valued their GP’s knowledge about them, their lives, and their illnesses.

3) The informants expressed a high degree of satisfaction with the quality of care received in general practice.

4) The informants expressed that geographical distance to the general practice was of minor importance to them.

**Conclusion:**

The patients perceived that the quality of the care and treatment they received were high following the transfer of COPD and T2D treatment to general practice. A strong, trusting relationship between the GP and the patient and the increased availability of the GP both contributed to their satisfaction with the GPs’ services.

## Background

Due to an ageing population with increasing morbidities and need for treatment, it was decided that the Danish healthcare system should undergo structural organisational changes [1]. Today, one-third of the Danish population is affected by one or more chronic disease, such as type 2 diabetes (T2D) and chronic obstructive pulmonary disease (COPD). By 2030, the number of patients with COPD and T2D will have increased to 60,000 and 200,000, respectively [2]. A significant number of these patients suffer from more than one chronic disease, leading to complex courses of treatment and contact with many different parts of the healthcare system [3, 4]. This impedes the patient’s experience of having coherent, high-quality, and well-coordinated treatment. Addressing these challenges requires development of a systematic and coordinated healthcare system [2, 5, 6]. There is a need for reorganisation of the healthcare system to ensure the most efficient use of resources. One way of achieving this goal is by mandating general practice to target resources to those patients who are most in need [7, 8]. To our knowledge, no other evaluations with the same scope as this study have been published. It is a political aim of the general practitioners (GPs) to become more responsible for the treatment and care of patients with chronic diseases to obtain the role of coordinator of the patient’s healthcare [9].

Part of this strategy is ensuring coherent patient pathways so that these patients are primarily managed in general practice and only the most complicated cases receive specialised hospital care [1]. Furthermore, it is assumed that GPs are familiar with their patients and have an understanding of each patient’s illnesses and treatments. Therefore, the GPs become the primarily professionals responsible for the care of the patients and function as gatekeepers and coordinators of specialised hospital care and municipal services [10, 11]. In Denmark, general practice is a medical speciality with its own theoretical and clinical curriculum lasting 5 years and is comparable with other medical specialties. A shift from symptom management and conventional treatment to a more systematic and holistic primary care approach is occurring in most European countries [12, 13]; however, to our knowledge, the patients’ experience-based perceptions of these organisational changes have not yet been published. Therefore, we aimed to explore COPD and T2D patients’ perceptions of the care delivered by their GPs. This knowledge is important for decision-makers so that they can adjust the system to support the aim of having the most efficient care possible.

### Setting

**Fig. 1:**
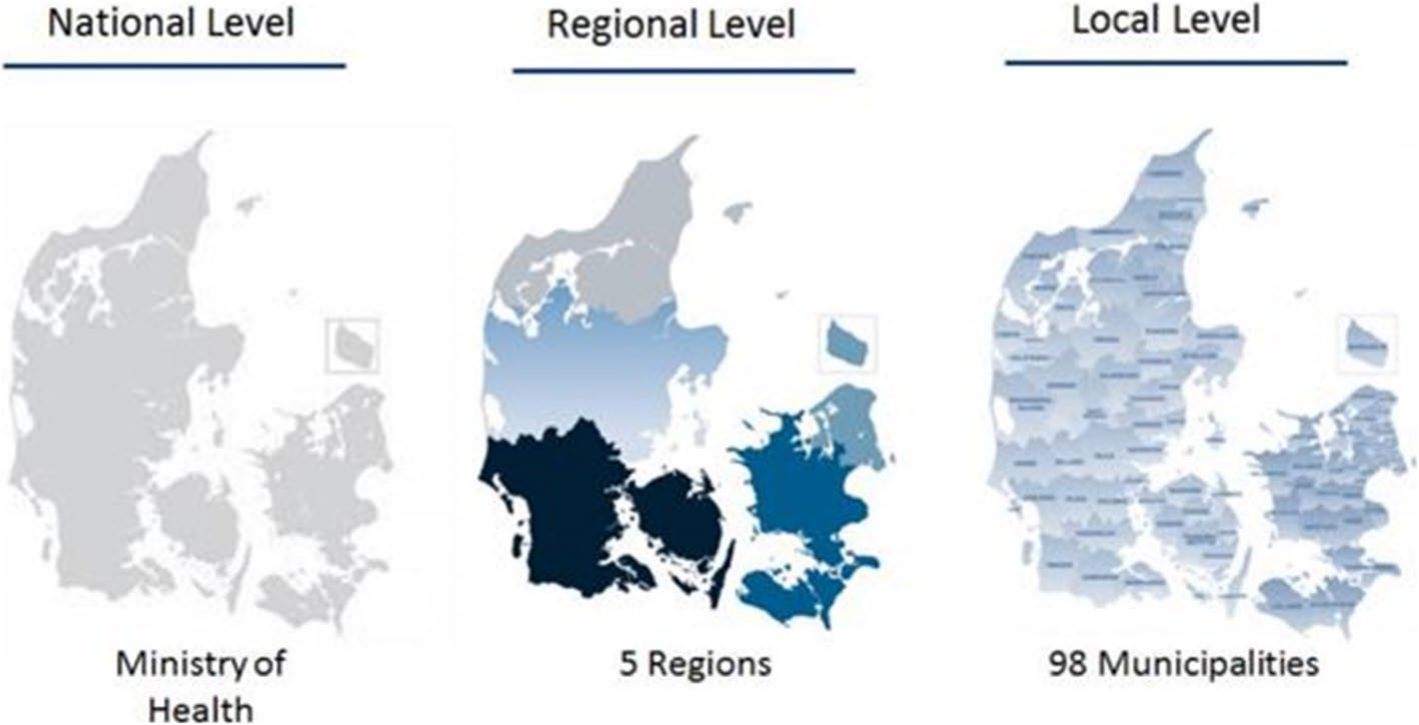
Organisation of the public sector in Denmark

The Danish healthcare system operates on three levels: national, regional, and primary. The national level consists of the Ministry of Health, which is responsible for governing the overall framework of the provision of healthcare and care of elderly. The regional level comprises five regions governed by elected councils; they are responsible for the hospitals and have an agreement about the primary healthcare provided by GPs. The hospitals are publicly owned, managed, and run by the regions. The GPs are independent private contractors who have a contract with the regions that is renewed every 3 years. The contract dictates that GPs receive a salary for consultations, laboratory work, and other predetermined functions relevant to their work with the patients they have enlisted. The contract ensures that the patients can consult their GP free of charge. The primary level concerns the 98 municipalities and each have their own elected council. The municipalities are responsible for several different services within primary care and the social sector, including rehabilitation, health promotion, and homecare/home nursing. The municipalities also co-finance regional rehabilitation and training provided by the region, which is a more specialised type of rehabilitation and training. The municipalities provide more general training, rehabilitation, and health interventions that are targeted toward the broader public. For a graphical overview, see Fig. 1.

## Method

We applied a descriptive qualitative design with individual semi-structured interviews with COPD or T2D patients whose GPs were responsible for their care. The data were generated between November 2019 and January 2020. Each interview was conducted at a location chosen by the patient, and most took place in the patient’s home or at the GP’s practice.

### Ethics

This study complied with ethical principles for medical research, as described in the Declaration of Helsinki. The study was reported to the Danish Data Protection Agency and the Institutional Review Board (10,791). The patients were informed of the purpose of the study both verbally and in writing, and verbal consent was obtained. The patients were informed that an audio recording of the interview would be made and that all identifying information would be deleted from the transcripts. The project was approved and financially supported by the Danish Regions. All data are stored in accordance with the European General Data Protection Regulations (GDPR).)

### Participants and recruitment

The snowball sampling method was used for the recruitment of patients. In this sampling method, gatekeepers of the healthcare system nominate potential patients for the research. In this study, GPs appointed their own patients with COPD or T2D who were (primarily) cared for in general practice. In addition, patients (informants) were recruited from patient associations. Informants were selected with the goal of including a variety of sexes, ages, and geographical locations; therefore, a purposive sampling procedure was applied. Three of the authors were responsible for the recruitment: Kim Lee (KL), Michael Marcussen (MM), and Signe Titlestad (SBT). The recruitment process ended when data saturation was reached.

### Interview and procedure

The individual semi-structured interviews were conducted by the authors KL, MM, and SBT. The authors’ backgrounds encompassed different disciplines, including occupational therapy, nursing, sports science, and general practice. The interview guide focused on four themes regarding patients’ perceptions of care in general practice: 1) proximity, 2) availability, 3) sense of security, and 4) quality. The interview guide was pilot-tested and revised prior to conducting the interviews. The pilots were included in the data because the patients met the inclusion criteria, and the interviews had the required level of quality. An audio recording of each interview was produced and transcribed for analysis. The transcriptions were produced verbatim in accordance with a transcription guide. The average duration of the interviews was 35 min. Respecting the informants’ workload, we chose not to ask them to further inspect or check and validate the transcribed material.

### Analysis

The data was analysed using systematic text condensation in four phases, as described by Malterud [14]. Systematic text condensation is a descriptive and explorative method of qualitative analysis. The procedure, according to Malterud, consists of the following steps:

- Step 1 total impression – Read the entire description in order to get a general sense of the whole statement.
- Step 2 identifying and sorting meaningful units – Go back to the beginning and readread through the text once more, with the specific aim of discriminating ‘“meaning units’units”, with a focus on the phenomenon being researched.
- Step 3 condensation—Once the meaning units have been delineated, inspectgo them all and expressexpresses the insight contained in the meaning units directly.
- Step 4 synthesising—Synthesises all of the transformed meaning units into a consistent statement regarding the subject’s experiences.

The text analysis was conducted by KL, MM, and SBT.

## Results

### Informant characteristics

In the study, 24 patients were individually interviewed, of which nine were diagnosed with COPD and 15 with T2D. Of these patients, 15 biologically identified as female and nine male, and they were all within the age range of 51–5186 years. Of the patients interviewed, eleven had either COPD or DM2, and 1 had both conditions.

Results are presented according to the themes guided by the analyses. The four main themes refer to the aim of the study: to explore the patients’ perceptions of the quality of care provided by their GP or general practice. The four core themes of this study were: 1) geographical distance, 2) availability, 3) sense of security, and 4) perceived quality of care.

### Geographical distance

Overall, the relationship with the GP and the patient’s perception of trust, sense of security, and quality of care were more important than the geographical distance to the GP’s clinic. One patient with COPD said:

‘It's not the distance that matters; it's about how you're treated as a person.’

Another informant emphasised this by stating that she would rather drive an extra 20 min to see her own GP rather see another GP at a clinic closer to her home. Both statements indicate the significant importance attached to the relationship between the GP and the informant. The meeting with the GP and the feeling of security are important to the informant, which highlights that the trust between the patient and their GP is highly important.

### Availability

The availability of the GP is also of significant importance to the informants. Availability is understood as the contact they have with their GP, either face-to-face, by telephone, or by e-mail. The informants’ perceptions of availability were closely tied to their experiences with being treated professionally and receiving adequate care, both of which increased the sense of trust in the treatment they received.

*‘If I call in the morning and ask, “Can I get hold of my own doctor or can he return my call?” and if he's preoccupied at the time with other patients, he usually calls me back shortly afterwards.’*

In general, the informants reported a high degree of GP availability in general practice, including the ability to receive an emergency appointment when needed. This availability had a major impact on how satisfied they were with the care that they received.

### Sense of security

A good relationship with the GP was considered especially important by the informants. This relationship building requires the GP to be thorough in their history taking and examinations, clearly explaining the disease and taking time to respond to the patient’s questions so that they understand the treatment and extent of their condition. It is essential for the informants to feel safe when they are talking with their GP. One informant said:

*‘I think it's important to feel safe and trust [in the GP], and I think it's important that you have the same doctor treating you so that you develop a relationship [between the patient and the GP] and ensure that you get the same treatment all the time [...] A GP can do that if they want to, whereas the doctors at the hospital can't have the same relationship with the patients.’*

It is important for the informants that the GP knows them well, including their background, relatives, and medical history. A close relationship between the GP and patient had immense significance for the patient’s perception of the quality of medical care that they received. A diabetic patient expressed:

‘I am a social person and need confirmation and support. And my doctor [GP] is good at that; I always feel like there's time for me—I feel that I am involved.’

It is important that the GP focuses on the patient’s trust and sense of security.

### Quality of the care

Quality of the care includes contact with healthcare professionals, communication, patient-involvement in treatment, and satisfaction with care. Therefore, it is important for the patients to be involved and take an active part in their own care. If patients are involved in their own care, they are more attentive to what the GP says; involvement in care affects how the patients listen to the GP’s recommendations on what to do and which treatment to choose.

When the GP involves a patient in decision-making regarding their care, the patient’s management becomes a collaboration between them and the GP, which contributes to strengthening the sense of trust. This is crucial for the informants’ experience of the quality of their care. Most informants choose to continue to remain under the care of the same GP, even if they move to another area. In addition, some informants prefer being treated by their GP, rather than being referred to a hospital. The informants believe that they receive the best treatment in general practice because of the trusting relationship that they have built with their GP:

‘I firmly believe that my GP does the best for me; she's not telling me to do it because it's the norm. She [the GP] sees me often and knows me and chooses what is best for me.’

This preference for their own GP suggests that most informants feel that they receive the best care possible in general practice. Moreover, their relationship with their GP is a key factor contributing to this perception.

## Discussion

The aim of our study was to explore patients’ perceptions of the quality of care they received in general practice. Our main findings were that most of the patients diagnosed with COPD or T2D perceived the relationship with their GP to be more important than the geographical distance to the GP's clinic. A good relationship between a patient and their GP is based on knowledge, trust, honesty, and support. Furthermore, the availability of the GP is a keystone for the patients, and it increased their satisfaction with the care that general practice could deliver. For the patients, it was of significant importance that their GP knew them and their medical history, which were essential factors for the patients’ experiences of the quality of the care they received from their GP.

To our knowledge, no previous published studies have explored COPD and T2D patients' perceptions on quality of care in general practice in Denmark. Our findings are in line with previous research, which demonstrated that the relationship between the patient and the GP was of great importance. This relationship is strengthened when the patient is primarily treated in general practice. Arreskov et al. (2019) found that the healthcare system needed the GP to be a coordinator of care, especially for patients with complex health problems who were contact with several healthcare providers. The GP has expertise as a medical care coordinator because of their knowledge of the patient's health problems and their unique relationship with the patient [15]. Likewise, a study conducted by Papp et al. (2014) showed that the patients’ perceptions of the quality of care they received in general practice was influenced by their relationship with the GP and their trust in their GP [16]. A similar conclusion was reached in a qualitative study of the relationship between the patient and their GP in primary care. In this study, Cocksedge et al. (2011) concluded that 1) these relationships were a routine part of general practice and were valued by both GPs and their patients, and 2) the GP–patient relationship was important in the management of patients with chronic and complex health problems [6]. The healthcare systems in Europe have recently faced major challenges caused by demographic changes, altered patterns of disease, and the decisions of political and private enterprises influencing the organisation of healthcare. The increase in the number of people suffering from chronic conditions and the transfer of care from secondary to primary providers has increased the number of tasks in general practice [17]. This study evaluated the perceived quality of care from a user perspective. Quality in general practice, according to the Danish College of General Practice, is composed of three elements: medical performance, organisational level, and patient-perceived quality [18]. This approach is in line with other studies exploring quality in general practice [19]. This study concludes that ‘countries with high level of general practice excel at empowering general practice to own the quality agenda’ [19], suggesting that future studies should aim to integrate the organisation (i.e., the other healthcare workers’ roles and importance) for the benefit of the patients. This work contributes to the understanding of quality of care in general practice.

### Limitations and strengths

A key strength of the study was that the characteristics of the participants varied widely, allowing findings to be applied to similar contexts in other (Western) countries. However, in such cases, culture, advancement, patient costs, and the structure of the healthcare system in the respective country must be considered.

The patients were recruited through general practices and local patient organisations, which may have led to underrepresentation of the sickest patients. The snowball sampling method may have led to inclusion of patients who were more resourceful and better at communicating their needs than the general population with COPD and T2D. However, this is a generic limitation that is often reported in qualitative studies investigating patients’ perceptions of their care [20]. The researchers’ preconceptions, knowledge from literature, and experiences with clinical work inevitably influenced the analysis and interpretation of the data. However, as the researchers had read all the transcripts and identified the core themes, we do not consider this as a limitation. A strength of the study is that multiple authors analysed the data and collaboratively discussed the results until they reached a consensus. The patients in the sample represented different perspectives on how they experienced their care and management in general practice; therefore, we consider the results valid.

## Conclusion

The patients perceived that, following the transfer of treatment for COPD and T2D to general practice, the quality of care and treatment was high. A strong, trusting relationship between the GP and the patient, and increased high availability of the GPs both contributed to the satisfaction with the GPs’ services. Furthermore, the availability of the patients’ GPs was of great importance for the patients and a significant contributing factor in their satisfaction with their care.

## Data Availability

To protect the anonymity of all the informants, the qualitative data used in this research cannot be made publicly available. Relevant data may be made available upon reasonable request from the corresponding author.

## Abbreviations

COPD: Chronic obstructive pulmonary disease
GP: General practitioner
T2D: Type 2 diabetes
